# Towards a molecular understanding of the apicomplexan actin motor: on a road to novel targets for malaria remedies?

**DOI:** 10.1107/S2053230X1500391X

**Published:** 2015-04-16

**Authors:** Esa-Pekka Kumpula, Inari Kursula

**Affiliations:** a Faculty of Biochemistry and Molecular Medicine, University of Oulu, PO Box 3000, 90014 Oulu, Finland; b Helmholtz Centre for Infection Research, Notkestrasse 85, 22607 Hamburg, Germany; c German Electron Synchrotron, Notkestrasse 85, 22607 Hamburg, Germany; d Department of Biomedicine, University of Bergen, Jonas Lies vei 91, 5009 Bergen, Norway

**Keywords:** malaria, actin polymerization, cytoskeleton, regulation, gliding motility, drug design, parasitology

## Abstract

Apicomplexan parasites are the causative agents of notorious human and animal diseases that give rise to considerable human suffering and economic losses worldwide. The most prominent parasites of this phylum are the malaria-causing *Plasmodium* species, which are widespread in tropical and subtropical regions, and *Toxoplasma gondii*, which infects one third of the world’s population. These parasites share a common form of gliding motility which relies on an actin–myosin motor. The components of this motor and the actin-regulatory proteins in Apicomplexa have unique features compared with all other eukaryotes. This, together with the crucial roles of these proteins, makes them attractive targets for structure-based drug design. In recent years, several structures of glideosome components, in particular of actins and actin regulators from apicomplexan parasites, have been determined, which will hopefully soon allow the creation of a complete molecular picture of the parasite actin–myosin motor and its regulatory machinery. Here, current knowledge of the function of this motor is reviewed from a structural perspective.

## Abstract 2

In this review, current structural understanding of the apicomplexan glideosome and actin regulation is described.

## 1. Introduction   

Apicomplexa are a vast group of ancient, unicellular eukary­otes. All of them are obligate parasites which are characterized by their unique apical organelles, and many are causative agents of notorious diseases of humans and domestic animals. Clinically, the most noteworthy species of this phylum are the intracellular *Plasmodium* spp. together with their cousin *Toxoplasma gondii*, which cause malaria and toxoplasmosis, respectively. Malaria is a devastating medical, economic and social problem in the poorest regions on Earth, causing hundreds of millions of infections and up to one million deaths annually, mainly among young children. *T. gondii* is an opportunistic pathogen that lies dormant in one third of the world’s population (Pappas *et al.*, 2009 ▶) but can become fatal for unborn foetuses and carriers with weakened immune systems, such as individuals suffering from acquired immunodeficiency syndrome or patients undergoing chemotherapy. Although the number of malaria deaths has been rapidly decreasing in the past decade, the emergence of already widespread drug resistance against artemisinin-based therapies casts a serious threat of a setback in efforts to eradicate this disease. Therefore, there is an urgent need for both novel drug targets and, preferably, vaccine development against malaria. Remedies against malaria may also provide clues to combat other apicomplexan diseases of medical or veterinary significance.

**Figure 1:**
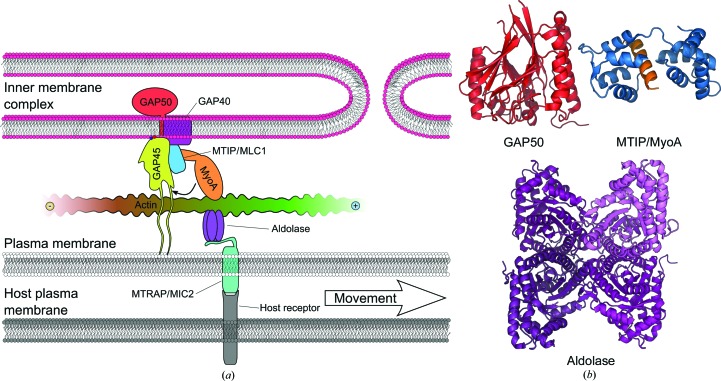
Schematic view of the apicomplexan pellicle with actin and the anchoring proteins. (*a*) The roles of the glideosome-associated proteins (GAP40, GAP45 and GAP50) and MyoA are inferred from data on *T. gondii*, while data also exist for *Plasmodium* spp. for the other components. Where two names are provided, the first one is always the *Plasmodium* protein and the second that from *T. gondii*. The small arrow indicates the direction of the MyoA power stroke, while the large arrow indicates the direction of parasite movement. The directionality of actin polymerization is indicated by + and − signs. (*b*) Structures of the *Plasmodium* GAP50 soluble domain (PDB entry 3tgh; Bosch *et al.*, 2012 ▶), *Plasmodium* MTIP in complex with a peptide from MyoA (PDB entry 4aom; Douse *et al.*, 2012 ▶) and *Plasmodium* aldolase (PDB entry 2pc4; Bosch, Buscaglia *et al.*, 2007 ▶).

Both *Plasmodium* and *T. gondii* have multi-phase life cycles involving a number of morphological transitions within different hosts. During certain stages the parasites move rapidly (1–3 µm s^−1^), making use of a unique form of motility referred to as gliding (Vanderberg, 1975 ▶), while in others they need to either penetrate a host cell or egress from one. These are active processes that have been tied to a parasite actin–myosin motor. However, the mechanism of force generation is not understood. The current working model of the motor postulates that very short actin filaments are used as linear tracks for myosin to move along. In this model, the actin–myosin motor is part of a mechanical complex termed the glideosome (Fig. 1 ▶) that links together the parasite plasma membrane and the inner membrane complex (IMC), a stacked membrane structure that provides structural strength and is also associated with invasion of and egress from host cells (Schatten *et al.*, 2003 ▶). However, recent work has shown that most of the glideosome components are at least to some extent redundant for both gliding motility and host-cell invasion (Andenmatten *et al.*, 2013 ▶; Egarter *et al.*, 2014 ▶). This raises the question whether additional alternative mechanisms to the linear motor exist or whether the whole model is incorrect.

While the central force-generating component in the linear model is the unconventional myosin MyoA, the real structural centerpiece of the system is actin. Actin is the most abundant protein inside eukaryotic cells and plays key roles in a plethora of structural and dynamic functions, including muscle contraction, cell-shape management and cell motility (Pollard & Cooper, 2009 ▶). Actin interacts with a multitude of different proteins, and in higher eukaryotes is regulated by at least 100 different actin-binding proteins (Dominguez, 2004 ▶). Apicomplexan actins are quite divergent at the amino-acid sequence level (Wesseling *et al.*, 1988 ▶) and also differ significantly from canonical actins in their functional properties (Skillman *et al.*, 2011 ▶; Vahokoski *et al.*, 2014 ▶). The apicomplexan pool of actin regulators is also much smaller, comprising only around ten actin-binding proteins (Sattler *et al.*, 2011 ▶), which can roughly be divided into two different groups: (i) proteins that bind actin in the monomeric, globular state (G-actin) and (ii) those that bind actin in the filamentous form (F-actin). Monomer binders function mainly by sequestering actin monomers and acting as nucleotide-exchange factors. Filament binders have various functions that are related to nucleation or stabilization of filaments or to the formation of higher-order structures.

In this review, we describe our current structural understanding of the apicomplexan actin–myosin motor, focusing on actin and actin-regulatory proteins from *Plasmodium* and *T. gondii*. In addition, we present our ideas on the feasibility of the parasite actin–myosin motor as a drug target.

## 2. Actin: the structural centrepiece   

Apicomplexan parasites express actins that, having less than 80% sequence identity to canonical actins of opisthokonts (animals and fungi) and plants, are among the most divergent members of this highly conserved protein family. Most apicomplexan genomes contain only one actin gene, with a notable exception being *Plasmodium* spp., which have two. The major isoform, actin I, from *Plasmodium* is a constitutively expressed, essential protein that is the most likely candidate to participate in the functions of the actin–myosin motor. In addition, actin I is also implicated in several other functions, including vesicle trafficking and endocytosis (Lazarus *et al.*, 2008 ▶; Smythe *et al.*, 2008 ▶), determining ring-stage morphology (Grüring *et al.*, 2011 ▶), the organization of genetic material in the nucleus (Zhang *et al.*, 2011 ▶) and apicoplast segregation (Andenmatten *et al.*, 2013 ▶). *Plasmodium* actin I is closely related to the single actins of other Apicomplexa. Actin II is specific to the mosquito stages of the *Plasmodium* life cycle and is as divergent from actin I as they both are from canonical actins. The functional assignment of actin II is so far somewhat ambiguous, but it plays important roles in a process called exflagellation during male gametocytogenesis (Deligianni *et al.*, 2011 ▶; Vahokoski *et al.*, 2014 ▶) as well as in ookinete formation and sporogony (Andreadaki *et al.*, 2014 ▶).

Apicomplexan actins have proven to be difficult to visualize *in vivo*, and it has not been clear what kind of structures they form in the cell. Until very recently, long filaments have not been visualized in apicomplexan parasites except for *Theileria* (Kühni-Boghenbor *et al.*, 2012 ▶). It has been proposed that the reasons for this include the high monomer:filament ratio and the short length, transient nature and rapid treadmilling of the filaments, as well as a lack of suitable tools for visualizing these unconventional actins (Schmitz *et al.*, 2005 ▶, 2010 ▶; Sahoo *et al.*, 2006 ▶). Recently, however, an actin cytoskeleton has been identified in *Plasmodium* gametocytes using super-resolution microscopy (Hliscs *et al.*, 2014 ▶). The filaments are mainly formed by actin I, form higher-order structures and are concentrated at the ends of the parasites, yet extend along the length of the elongated gametocyte, following the microtubules. Surprisingly, these actin filaments are not located in the sub-alveolar space between the plasma membrane and the IMC, but rather on the cytoplasmic side of the IMC. These findings imply that with the development of high-resolution imaging systems it may also be possible to visualize actin filaments in other apicomplexan parasites and different developmental stages.

**Figure 2:**
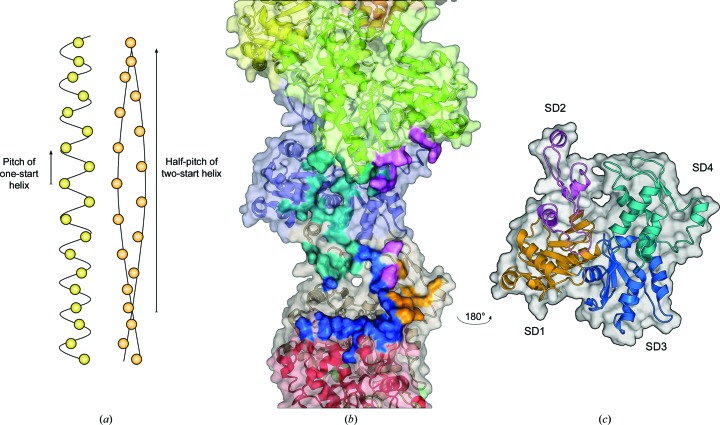
Structure of an actin filament. (*a*) Schematic representation of the one-start and two-start helices and their respective pitches or half-pitches, adapted from Vahokoski *et al.* (2014 ▶). (*b*) Structure of an α-actin filament (PDB entry 3g37; Murakami *et al.*, 2010 ▶) with one of the monomers removed. Intense surface colours indicate contact regions with the subdomains of the removed monomer (SD1, yellow; SD2, magenta; SD3, blue; SD4, turquoise). Lighter surface colours denote different monomers in the filament. (*c*) The removed actin monomer rotated by 180° to show the filament-facing side of the monomer.

The first structural studies of apicomplexan actin filaments were undertaken on endogenous *Plasmodium* actin purified from merozoites using atomic force microscopy (Schmitz *et al.*, 2010 ▶). More recently, we characterized the filaments formed by both *Plasmodium* actins using cryo-EM (Vahokoski *et al.*, 2014 ▶). Because purified actin I alone does not form filaments that are long enough for structural characterization, jasplakino­lide was used to stabilize the filaments. Another widely used actin filament-stabilizing agent, phalloidin, has low affinity for actin I (Schmitz *et al.*, 2010 ▶), suggesting structural differences in the binding site. *Plasmodium* actin I filaments clearly differ from α-actin filaments, whereas actin II forms filaments similar to canonical F-actin. A helical actin filament can be described using two values that describe the pitch of the one-start helix and the half-pitch of the two-start helix (Fig. 2 ▶). The pitch of the one-start helix is identical in both canonical and apicomplexan actins (60 Å), but the half-pitch of the two-start helix is significantly longer in *Plasmodium* actin I: ∼400 Å compared with ∼360–370 Å in canonical actins (Fujii *et al.*, 2010 ▶; Schmitz *et al.*, 2010 ▶; Vahokoski *et al.*, 2014 ▶). This structural change is owing to an altered helical rotation (that is, the rotation that takes place from one monomer to the next in the one-start helix) rather than helical rise. In other words, the monomer density within a given length of filament is the same between these filaments, but the relation of the monomers to one another is different. The twist of −167.5° in the *Plasmodium* actin I filament is different to the canonical actin filament, where the twist is −166.6° (Fujii *et al.*, 2010 ▶). Interestingly, the direction of the change is the opposite compared with the change in twist induced by the binding of the filament-severing protein cofilin, which causes an unwinding of the filament by 5° (McGough *et al.*, 1997 ▶).

**Figure 3:**
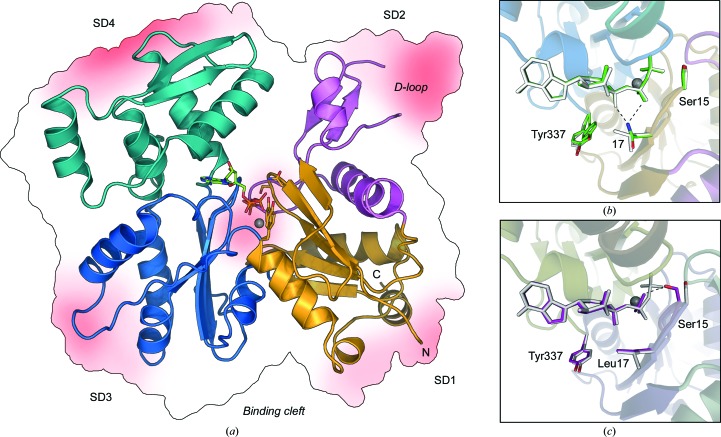
Structure of monomeric actin. (*a*) Monomeric *P. falciparum* actin I (PDB entry 4cbu; Vahokoski *et al.*, 2014 ▶). The regions with the largest differences from α-actin (PDB entry 1eqy; McLaughlin *et al.*, 1993 ▶) are indicated with a red hue in the background. Colour-coding of the protein is as in Fig. 2 ▶. ATP is shown as sticks and the bound Ca^2+^ ion is shown as a grey sphere. The subdomains are numbered and the N- and C-termini as well as the general actin-binding protein binding cleft and the D-loop are labelled. (*b*) Comparison of the active-site details of *P. falciparum* actin I (green) and α-actin (grey). Tyr337 of actin I is in a double conformation. Residue 17 is an asparagine in actin I and a leucine in α-actin. Residue numbering follows that of *P. falciparum* actin I. (*c*) Conformational changes in the active site of α-actin upon ATP hydrolysis. ATP state, PDB entry 1nwk (Graceffa & Dominguez, 2003 ▶; grey); ADP state, PDB entry 1j6z (Otterbein *et al.*, 2001 ▶; magenta). Residue 17 is a leucine in both structures. For clarity, the residue numbering follows that of *P. falciparum* actin I, as in (*b*).

Actin monomers share a four-subdomain fold with sugar kinases and Hsp70 proteins (Bork *et al.*, 1992 ▶). The sub­domains in monomeric actin are arranged in a slightly crooked horseshoe shape, with an active site in the middle containing an adenosine nucleotide (ATP or ADP) and a bound metal cation (Fig. 3 ▶; Kabsch *et al.*, 1990 ▶). Additional notable structural elements in the actin structure include: (i) the DNase I binding loop (D-loop), a flexible loop in subdomain 2 that is inserted inside the actin filament upon polymerization, mediating protomer interactions (Oda *et al.*, 2009 ▶; Murakami *et al.*, 2010 ▶; Figs. 2 ▶ and 3 ▶ *a*) and (ii) a hydrophobic cleft between subdomains 1 and 3, which is the binding site for most of the monomer-binding proteins (Dominguez, 2004 ▶; Fig. 3 ▶ *a*). *Plasmodium* actins share their overall fold with all other actins. However, various small differences can be linked to the different polymerization properties of the parasite actins (Vahokoski *et al.*, 2014 ▶). Considering the proposed unstable nature and fast polymerization/depolymerization kinetics of the actin I filaments, it is not surprising that the largest backbone root-mean-square differences compared with canonical actins are found in the areas involved in protomer contacts in the filament (Fig. 3 ▶ *a*).

## 3. Peculiar behaviour of apicomplexan actins   

The fundamental property of actin enabling most of its cellular functions is its ability to form dynamic structures with a defined polarity. Upon polymerization, the canonical actin protomer assumes a flattened, catalytically active conformation (Oda *et al.*, 2009 ▶), leading to ATP hydrolysis in the filament. The hydrolysis of ATP and the subsequent release of free phosphate lead to a further conformational change that in turn destabilizes the protomer contacts inside the filament. This eventually leads to dissociation of ADP–actin monomers from the ‘aged’ end of the filament, whereas rapid polymerization continues by the addition of ATP-loaded monomers to the fast-growing end.

Canonical actins polymerize efficiently at high ionic strength and in the presence of Mg^2+^ ions. Apicomplexan actins can also be polymerized in similar conditions, but even after high-speed ultracentrifugation the ratio of actin in the pellet and supernatant fractions is close to one for *Plasmodium* actin I and *T. gondii* actin, whereas actin II can be polymerized nearly completely (Skillman *et al.*, 2011 ▶, 2013 ▶; Ignatev *et al.*, 2012 ▶; Vahokoski *et al.*, 2014 ▶). This is in line with estimates that the majority of apicomplexan actin remains monomeric *in vivo* (Field *et al.*, 1993 ▶; Dobrowolski *et al.*, 1997 ▶). Light-scattering-based assays of *T. gondii* actin suggest that there is no lag phase as typically associated with a nucleation/elongation type of filament growth (Skillman *et al.*, 2013 ▶). Using sedimentation assays, it was also found that *T. gondii* actin lacks an apparent critical concentration for polymerization, one of the central parameters of canonical actins. These findings led to the proposal of an isodesmic, nucleation-independent polymerization model (Skillman *et al.*, 2013 ▶). On the other hand, *Plasmodium* actins spontaneously form small oligomers within hours, which we could link to the hydrolysis of ATP, which also readily occurs in the monomeric state in *Plasmodium* actins (Vahokoski *et al.*, 2014 ▶). ADP–actin is also inherently less stable and thus more prone to aggregation (Pivovarova *et al.*, 2010 ▶), a process that complicates the interpretation of light-scattering experiments. Clearly, further work is required to elucidate the polymerization mechanism of apicomplexan actins and the structure and purpose of the ADP-induced oligomers. Direct evidence of similarity in the polymerization behaviour of the different parasite actins is also still lacking, so caution must be taken with any inferences.

The nucleotide-hydrolysis rate, measured indirectly as the rate of phosphate release that is rate-limiting in canonical actins, is higher in *Plasmodium* actins, especially in actin I, compared with α-actin (Vahokoski *et al.*, 2014 ▶). Differences in the reaction rate are likely to occur owing to two structural reasons: (i) residue-level differences in the active site that alter the mechanistic details of hydrolysis and (ii) subtle changes in tertiary structure that change the orientations of subdomains relative to each other. One clear residue-level difference in the active site of actin I compared with most other actins is residue 17, which is an asparagine in actin I but is either a leucine or a methionine in other actins, including that from humans. This asparagine is in close proximity to the β-phosphate of ATP in the crystal structure (Fig. 3 ▶ *b*) and thus may play a pivotal role in the catalytic mechanism. This difference in the active site calls for more detailed structural and enzymological studies and may open possibilities for the design of specific compounds that bind to the active site of apicomplexan actins.

The larger structural factors affecting the hydrolysis rate are the relative positions of the so-called outer domain (sub­domains 1 and 2) and inner domain (subdomains 3 and 4) of the actin monomer and the conformation of the D-loop. A striking increase in the hydrolysis rate could be observed for a chimeric mutant in which the D-loop of *Plasmodium* actin I was replaced with that of α-actin (Vahokoski *et al.*, 2014 ▶). One of the more interesting single residues in this region is Phe53 in actin I, which is a Tyr in almost all other actins. The tyrosine residue is a common phosphorylation site, and mutating it to a phenylalanine in *Dictyostelium discoideum* actin causes only modest changes in function (Liu *et al.*, 2010 ▶), but obviously prevents control by phosphorylation. Phosphorylation of the tyrosine in *D. discoideum* actin results in a slight stabilization of the D-loop conformation as well as inhibition of nucleotide exchange and decreased filament stability (Liu *et al.*, 2006 ▶, 2010 ▶). *Plasmodium* actin II, which forms stable long filaments, has a tyrosine residue at this position. Interestingly, an F54Y mutant of actin I shows hydrolysis rates comparable to α-actin in the Ca^2+^-bound state, while performing identically to the wild type in the Mg^2+^-bound state (Vahokoski *et al.*, 2014 ▶).

## 4. Small monomer-binding nucleotide-exchange factors   

Apicomplexan genomes encode three classes of proteins that bind to monomeric actin in opisthokonts and influence the rate of nucleotide exchange in the ATP/ADP-binding pocket of actin. As it seems that at least *Plasmodium* actins also readily hydrolyze ATP in the monomeric form, nucleotide-exchange activity may be of special importance among the monomer-binding proteins in these parasites.

### 4.1. Profilin   

**Figure 4:**
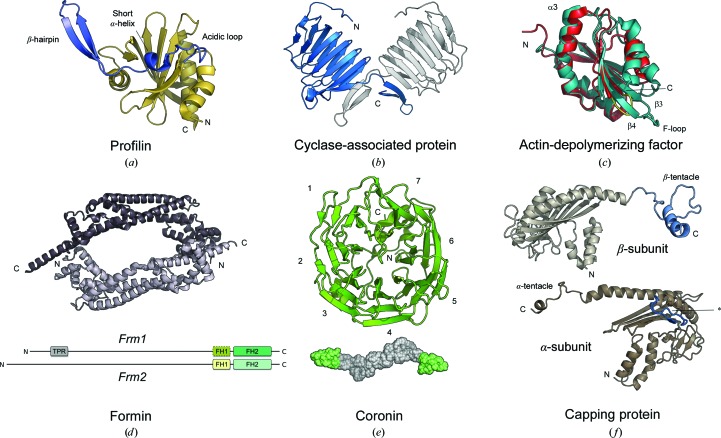
Apicomplexan actin-regulatory proteins. (*a*) *P. falciparum* profilin (PDB entry 2jkg; Kursula, Kursula, Ganter *et al.*, 2008 ▶). The Apicomplexa-specific region is indicated in blue. (*b*) *Cryptosporidium parvum* CAP (PDB entry 2b0r; Hliscs *et al.*, 2010 ▶). The two monomers of the dimer are shown in blue and grey. (*c*) *P. falciparum* ADF1 (PDB entry 2xf1; Singh *et al.*, 2011 ▶; red) and *P. berghei* ADF2 (PDB entry 2xfa; Singh *et al.*, 2011 ▶; turquoise). The very short F-loop of ADF1 is indicated in yellow. (*d*) *Mus musculus* mDia1 FH2 domain crystal structure (PDB entry 1v9d; Shimada *et al.*, 2004 ▶), with a schematic view of the domain architecture of *Plasmodium* formins 1 and 2. The FH1 domain of formin 1 is indicated with a dashed line to denote its putative nature. (*e*) *T. gondii* coronin WD40 domain (PDB entry 4ozu; Salamun *et al.*, 2014 ▶) and a SAXS model (Salamun *et al.*, 2014 ▶) of the full-length protein, with green indicating the location of the WD40 domains in the dimer. The β-propeller blades are numbered from the N-terminus to the C-terminus. (*f*) Homology models of the *P. falciparum* CP α- and β-subunits based on the structure of *Gallus gallus* CapZ (PDB entry 1izn; Yamashita *et al.*, 2003 ▶). The 23-residue insertion in the α-subunit (*) and the extended β-tentacle region are indicated in blue.

Profilins are small actin monomer-sequestering proteins that facilitate nucleotide exchange in the actin monomer and accelerate polymerization in concert with formins by providing readily polymerizable actin monomers to the fast-growing end of the filament (Kovar *et al.*, 2006 ▶). The former function has been related to the state of the nucleotide-binding cleft in profilin-bound actin, which is considered to be ‘open’ to favour the exchange of the nucleotide in the active site (Porta & Borgstahl, 2012 ▶). The latter effect is a consequence of the interaction of profilins with multiple proline-rich repeats in formins, which nucleate actin filaments and stay bound to the growing barbed end (Otomo, Tomchick *et al.*, 2005 ▶). Apicomplexa encode single profilins that contain large sequence insertions compared with higher eukaryotic profilins. Three-dimensional structures have been determined for the *P. falciparum* and *T. gondii* profilins (Kursula, Kursula, Ganter *et al.*, 2008 ▶; Kucera *et al.*, 2010 ▶), and both show that these insertions form a parasite-specific structural motif consisting of an acidic loop of variable length, a short α-helix and a long β-hairpin extension (Fig. 4 ▶ *a*). Despite the large insertion, the parasite profilins share with their canonical counterparts a conserved core of a seven-stranded β-sheet sandwiched between two α-helices on each side. *Plasmodium* profilin binds actin, proline-rich peptides and phosphatidyl­inositide lipids (Kursula, Kursula, Ganter *et al.*, 2008 ▶), thereby fulfilling the key functional characteristics of profilins. On the contrary, *T. gondii* profilin apparently lacks the ability to bind proline-rich peptides and a range of phospholipids (Kucera *et al.*, 2010 ▶). Both parasite profilins are weak sequesterers of mammalian actins (Kursula, Kursula, Ganter *et al.*, 2008 ▶; Kucera *et al.*, 2010 ▶).

The actin-binding mode of apicomplexan profilins has not been resolved, but the β-hairpin extension seems to be important for binding (Kursula, Kursula, Ganter *et al.*, 2008 ▶; Kucera *et al.*, 2010 ▶), which makes this interface unique among all known profilin–actins and renders the interaction a putative target for structure-based drug design. The effect of *Plasmodium* profilin on nucleotide exchange is not known. *T. gondii* profilin, however, curiously inhibits ATP exchange on rabbit muscle actin (Kucera *et al.*, 2010 ▶). As the current work on the binding of apicomplexan profilins to actin has been performed using heterologous actins, it is very important now to use the *Plasmodium* and *T. gondii* actins that have recently been produced as recombinant proteins (Skillman *et al.*, 2011 ▶; Ignatev *et al.*, 2012 ▶; Bhargav *et al.*, 2013 ▶) to shed light on the true affinities, binding modes and biochemical activities of the parasite profilins on the respective parasite actins.

The *P. falciparum* profilin crystal structure has also been determined in the presence of an octaproline peptide (Kursula, Kursula, Ganter *et al.*, 2008 ▶). Despite notable differences in the residues involved in peptide binding, the binding mode closely resembles that of canonical profilin–peptide complexes. Unlike other profilins, the *Plasmodium* profilin C-terminal α-helix is not involved in interactions with the peptide. Possibly owing to the lack of this interaction and differences in some of the aromatic peptide-binding residues, the affinity of *Plasmodium* profilin for octaproline is lower than in mammalian profilins (Kursula, Kursula, Ganter *et al.*, 2008 ▶). Apicomplexan formins contain only rudimentary FH1 domains and, for example, *T. gondii* profilin was shown not to bind to a peptide derived from the *T. gondii* formin 2 (Kucera *et al.*, 2010 ▶). Thus, it is not clear whether the cooperative mechanism of actin-filament elongation by profilin and formins is conserved in Apicomplexa.

The different apicomplexan profilins crudely share the same parasite-specific structural features. However, the sequence conservation of the insertions within the phylum is low, and the profilins from *Plasmodium* and *T. gondii* also seem to have at least partly different functional properties. A notable difference between the apicomplexan profilins is the length of the acidic loop, which is longer in *Plasmodium* than in other Apicomplexa. However, the *T. gondii* loop is more acidic, with four aspartate residues in a row. This loop resides far from the actin-binding site and is also not involved in proline-rich peptide binding, but has been shown to be responsible for a toll-like receptor 11 (TLR11)-mediated interleukin IL-12 immune response in mice (Kucera *et al.*, 2010 ▶). Previously, *T. gondii* profilin was identified as the first ligand for mouse TLR11 (Yarovinsky *et al.*, 2005 ▶). The *Plasmodium* profilin acidic loop causes a reduced immune response, while, on the other hand, insertion of the *T. gondii* profilin acidic loop with or without the β-hairpin motif into yeast profilin is sufficient to induce IL-12 production in mice (Kucera *et al.*, 2010 ▶). The structural basis for TLR11–profilin recognition is not known. Curiously, humans express no functional TLR11 (Lauw *et al.*, 2005 ▶), so the TLR11–IL-12 pathway is likely not to be relevant in human infection.

While the most prominent role of profilins is in the regulation of actin dynamics, it seems that they have multifaceted functions in the pathogenesis of apicomplexan parasites. Profilin is essential for the survival of *P. falciparum* (Kursula, Kursula, Ganter *et al.*, 2008 ▶) and is indispensable for gliding, host-cell invasion and egress, and virulence of *T. gondii* (Plattner *et al.*, 2008 ▶). Yet, canonical human profilin can fulfill the essential functions in *Plasmodium* (Kursula, Kursula, Ganter *et al.*, 2008 ▶). So, what is the reason for the presence of such structurally divergent profilins in these parasites and are these profilins results of divergent or convergent evolution? In particular, the role of parasite profilins in the host immune response, and possibly immune evasion, warrants further investigation.

### 4.2. Cyclase-associated protein   

Cyclase-associated proteins (CAPs) are a group of multi-domain proteins that are named after their association with adenylate cyclase, an enzyme widely implicated in cellular signalling owing to the reaction that it catalyzes: the generation of cAMP from ATP (Fedor-Chaiken *et al.*, 1990 ▶; Field *et al.*, 1990 ▶). In the yeast CAP Srv2, the N-terminal domain is required for the adenylate cyclase interaction (Gerst *et al.*, 1991 ▶) as well as for the binding of cofilin–actin complexes (Moriyama & Yahara, 2002 ▶; Quintero-Monzon *et al.*, 2009 ▶). The C-terminal region sequesters G-actin and facilitates nucleotide exchange from ADP to ATP. The region connecting these terminal domains in Srv2 contains a WH2 motif flanked by proline-rich segments that bind profilin (Bertling *et al.*, 2007 ▶). Apicomplexan CAPs are much shorter than their orthologues in other species. *Plasmodium* CAP lacks the N-terminal domain as well as the connecting middle region found in other CAPs. However, *T. gondii* and *Cryptosporidium parvum* CAPs contain a short N-terminal extension with low sequence homology to the WH2 segment in other CAPs (Hliscs *et al.*, 2010 ▶; Makkonen *et al.*, 2013 ▶).

The *C. parvum* CAP, much like the C-terminal domain of Srv2, is a V-shaped homodimer composed of two right-handed β-helices intertwined in the C-terminus *via* a domain swap of single β-strands (Fig. 4 ▶ *b*; Hliscs *et al.*, 2010 ▶). The β-helices feature five stacks of hydrophobic residues and one stack of Cys/Ser residues. A difference from other C-terminal CAP structures is that one of the hydrophobic stacks in *C. parvum* CAP is composed of a hydrophobic and a hydrophilic (Cys-Ser-Cys) segment. This hydrophilic half-segment is evidently not conserved in *T. gondii* CAP (Lys-Val-Leu), while being somewhat conserved in *Plasmodium* CAPs (Glu/Gly-Thr-Cys). The *Plasmodium* and *Cryptosporidium* CAPs characterized to date bind actin monomers and catalyze nucleotide exchange. However, the exact actin-interacting sites are not known. As the short apicomplexan CAPs lack the domains interacting with multiple binding partners, it seems that their function is limited to the reloading of actin monomers with ATP. In genome-scale mRNA-based expression profiling of *P. berghei* (Otto *et al.*, 2014 ▶) and *P. falciparum* (Otto *et al.*, 2010 ▶), expression of CAP was found to peak in the late intraerythrocytic stages, increasing steadily after invasion to peak at 22 h post-invasion. This would suggest a specialized function of CAP during the intraerythrocytic stages and/or egress, perhaps by sequestering free actin monomers to prepare for the burst of movement during egress.

### 4.3. Actin-depolymerizing factors   

Actin-depolymerizing factors (ADFs) belong to the ADF/cofilin superfamily of proteins, which have a complex role in determining filament dynamics and are controlled by a complex regulatory network in higher eukaryotes (Van Troys *et al.*, 2008 ▶). The ADF/cofilin proteins bind and possibly sequester actin monomers, classically preferring the ADP-bound state of actin and inhibiting nucleotide exchange, thereby also inhibiting the incorporation of the monomers released from the pointed end back to the barbed end (Carlier *et al.*, 1997 ▶; Suarez *et al.*, 2011 ▶). However, ADF/cofilins also bind to filaments with concentration-dependent effects (Andrianantoandro & Pollard, 2006 ▶). There are two models of how ADF/cofilins affect F-actin: the disassembly model, in which the ADF/cofilins increase the rate of dissociation of actin monomers from the pointed end, and the severing model, in which they chop filaments into smaller pieces (Carlier *et al.*, 1999 ▶; Ichetovkin *et al.*, 2002 ▶; Pavlov *et al.*, 2007 ▶). While both result in shorter filaments, both may also increase the rate of polymerization. Increased depolymerization at the pointed end results in a higher number of free monomers to be added back to the barbed end. Severing of filaments in turn generates new free barbed ends. At moderate concentrations ADF/cofilins stabilize actin filaments by saturating the filament (termed ‘decoration’), while at very high concentrations ADF/cofilin-mediated nucleation can occur (Andriananto­andro & Pollard, 2006 ▶). The binding of ADF to an actin filament induces an unwinding of approximately 5° of the actin helix, which is presumably the cause of both depolymerization and severing (McGough *et al.*, 1997 ▶; Galkin *et al.*, 2011 ▶).

The ADF/cofilin core structure, or the so-called ADF-homology domain (Fig. 4 ▶ *c*), is composed of a central mixed β-sheet (β1–β6) that is sandwiched between two longer α-helices (α1 and α3) on one side and two shorter α-helices (α2 and α4) on the other. Between β-strands 3 and 4 is a region termed the F-loop that is predicted to be important for F-actin binding based on models of cofilin-decorated actin filaments (Galkin *et al.*, 2011 ▶). Besides the F-loop, helices α4 and α1 as well as the N-terminus participate in interactions with the actin filament (Galkin *et al.*, 2011 ▶). The G-actin binding site, as seen in the structure of the twinfilin C-terminal ADF-homology domain in complex with actin, includes the ADF N-terminus that interacts with the C-terminus of actin, the N-terminal part of ADF α3 that inserts itself between subdomains 1 and 3 of actin and the loop before the C-terminal α4 of ADF that interacts with subdomain 3 of actin (Paavilainen *et al.*, 2008 ▶).

*Plasmodium* spp. express two ADF isoforms that share the core fold with canonical ADF/cofilins but also have substantial differences compared with each other and with other family members (Fig. 4 ▶ *c*). The most striking difference between ADF1 and ADF2 of *Plasmodium* is the length of the F-loop, which is nine residues shorter in ADF1 (Singh *et al.*, 2011 ▶; Wong *et al.*, 2011 ▶) compared with ADF2. Other differences include missing hydrophobic residues in α3 implicated in G-actin binding (Paavilainen *et al.*, 2008 ▶; Singh *et al.*, 2011 ▶) and a shorter C-terminus in ADF1 (Singh *et al.*, 2011 ▶). The differences in the F-loop seem to translate into functional differences between the ADF isoforms. ADF1, with a very short F-loop, does not show high-affinity binding to filaments (Schüler *et al.*, 2005 ▶), whereas contradictory findings have been made regarding its ability to sever filaments (Singh *et al.*, 2011 ▶; Wong *et al.*, 2011 ▶, 2014 ▶). The filament dimensions, particularly the pitch of the two-start helix and thus the helical twist, were found to be unaffected by the presence of ADF1, suggesting that severing does not necessarily require the strain induced by the change in helical symmetry reported for canonical ADFs (Wong *et al.*, 2014 ▶). Furthermore, a non­canonical binding site on F-actin has been proposed based on chemical cross-linking coupled to mass spectrometry. ADF1 seems to bind to the outer face of single actin monomers in the filament, as opposed to the canonical binding site located between monomers *n* and *n* + 2 (Wong *et al.*, 2014 ▶). In the same study, a noncanonical G-actin binding mode was also proposed for ADF1. While these findings on parasite ADFs with canonical actin propose possibly unique mechanisms of ADF/cofilin function in Apicomplexa, the biological significance needs verification with the most likely true binding partner: *Plasmodium* actin I. This is especially important as the difference in filament twist could mask crucial contact residues in canonical actins, while inducing a preference for an otherwise insignificantly populated binding site.

*Plasmodium* ADF2 can bind to and sever canonical actin filaments, as expected based on its conserved structure (Singh *et al.*, 2011 ▶; Wong *et al.*, 2011 ▶). Both ADF2 (Doi *et al.*, 2010 ▶) and actin II (Andreadaki *et al.*, 2014 ▶) are insect-stage specific in the *Plasmodium* life cycle. This, together with the fact that actin II filaments structurally resemble canonical actins, raises the question whether ADF2 could interact with actin II *in vivo*, but so far experimental evidence supporting this is lacking.

*T. gondii* has a single ADF (*Tg*ADF) that closely resembles *Plasmodium* ADF1. It sequesters α-actin monomers and binds α-actin filaments with low affinity, causing severing (Mehta & Sibley, 2010 ▶). The interaction of *Tg*ADF with *T. gondii* actin filaments has been studied (Mehta & Sibley, 2010 ▶). Unlike with α-actin filaments, *Tg*ADF did not stably associate with *T. gondii* actin filaments, while *Schizosaccharomyces pombe* cofilin associated with both filaments under similar conditions (Mehta & Sibley, 2010 ▶). Also, unlike *Plasmodium* ADFs, *T. gondii* ADF was shown to inhibit nucleotide exchange in a manner similar to classical ADF/cofilins (Mehta & Sibley, 2010 ▶). The solution structure of *T. gondii* ADF reveals a fold similar to *Plasmodium* ADF1, with a very short F-loop (Yadav *et al.*, 2011 ▶).

In conclusion, many aspects of apicomplexan ADFs challenge the classical view of ADF/cofilin functions. The intrinsically fast treadmilling of parasite actin filaments alone questions the need for the action of classical ADFs (Schmitz *et al.*, 2005 ▶). The reversed activity on nucleotide exchange places this requirement even further into questionable territory (Singh *et al.*, 2011 ▶). In particular, the possibly novel mode of ADF1 binding to actin (Wong *et al.*, 2014 ▶) exemplifies the need for further research into this particular area of actin regulation in Apicomplexa and also higher eukaryotes, and may render the *Plasmodium* ADF1–actin interaction as an attractive drug target.

## 5. Large filament-binding proteins   

The unconventional filament structure and dynamics of *Plasmodium* actin I and possibly other apicomplexan actins may pose special requirements for filament-binding proteins. The filament binders can be roughly divided into two categories: proteins binding to filament ends and those binding to the sides of filaments. The end-binding proteins typically cap filaments, preventing both the gain and loss of monomers from the ends, and may also act as nucleators/processive cappers, accelerating the rate of polymerization. Proteins binding to the sides along the filament length may stabilize or destabilize filaments and induce higher-order structures *via* cross-linking or bundling. Taking into account the inherent instability of the parasite actin filaments, one may assume that nucleation and stabilization of filaments would be of special importance among the parasite F-actin-binding proteins. Yet, most of the classical nucleators as well as stabilizing and cross-linking factors are actually missing in apicomplexan parasites. Also, no pointed-end binding proteins have been identified.

### 5.1. Formins   

Formins are the single most likely candidate for an actin-filament nucleator in Apicomplexa. The members of the formin superfamily are large multi-domain proteins, classically of some 1000–1700 amino acids in length. The best characterized classical formin is the mammalian mDia1 along with the yeast homologue Bni1p, which are also structurally the most studied formins (Shimada *et al.*, 2004 ▶; Lammers *et al.*, 2005 ▶; Otomo, Otomo *et al.*, 2005 ▶; Otomo, Tomchick *et al.*, 2005 ▶; Otomo *et al.*, 2010 ▶; Rose *et al.*, 2005 ▶; Nezami *et al.*, 2006 ▶, 2010 ▶; Kursula, Kursula, Massimi *et al.*, 2008 ▶). For a review of the different classes of formins in humans, see, for example, Schönichen & Geyer (2010 ▶). Formins are characterized by two formin homology (FH) domains. The usually C-terminal actin-binding FH2 domain is the defining structural characteristic of the superfamily. It forms a ring-like homodimeric structure (Fig. 4 ▶ *d*) that binds at the barbed end and nucleates actin filaments even in the absence of other formin domains. However, a block in dimerization brought about by the removal of an N-terminal subdomain (the lasso region) causes the FH2 domain to inhibit filament growth instead (Pruyne *et al.*, 2002 ▶; Shimada *et al.*, 2004 ▶). The FH1 domain, which is usually located N-terminal to the FH2 domain, is characterized by a variable number of proline-rich stretches that bind profilin, serving as a local store of profilin–actin complexes that can be fed to the growing filament (Kovar *et al.*, 2006 ▶; Kursula, Kursula, Massimi *et al.*, 2008 ▶). Other domains typically found in formins include the GTPase-binding domain (GBD), the diaphanous autoinhibitory domain (DAD) and the diaphanous inhibitory domain (DID or FH3). However, apicomplexan formins lack any apparent sequences corresponding to these regulatory domains.

*Plasmodium* spp. have two cytosolic formins (formins 1 and 2; Baum *et al.*, 2008 ▶), as well as a nucleus-associated formin-like protein MISFIT (Bushell *et al.*, 2009 ▶). *Plasmodium* formins are much larger than their canonical counterparts, consisting of ∼2000–3000 amino acids (Fig. 4 ▶ *d*). Both formins 1 and 2 contain a C-terminal conserved FH2 domain and an upstream sequence which is thought to correspond to the classical FH1 domain, despite a very low proline content. Besides the FH2 and the putative FH1 domains, the only region displaying significant homology to any known protein is the tetratricopeptide repeat (TPR) sequence in the N-terminal portion of formin 1. TPR motifs are classically scaffolding units associated with the formation of multi-protein complexes.

Like *Plasmodium*, *T. gondii* encodes two cytosolic formins (Daher *et al.*, 2010 ▶) and a nuclear formin (Daher *et al.*, 2012 ▶), which however has no apparent sequence homology to the *Plasmodium* protein MISFIT and is non-essential for the parasite. The cytosolic formins of *T. gondii* are even larger than those of *Plasmodium*, with lengths of close to 5000 amino acids. Despite the size difference, the functions of these proteins seem to be quite similar. Formins 1 and 2 of Apicomplexa are, owing to their cytosolic localization, the most likely candidates for involvement in the actin–myosin motor.

In terms of nucleating activity, formin 1 of either *Plasmodium* or *T. gondii* is slightly more efficient than the corresponding formin 2, with maximal nucleation capacity reached in the submicromolar to low micromolar range as shown by assays with rabbit skeletal muscle actin (Baum *et al.*, 2008 ▶; Daher *et al.*, 2010 ▶). However, when analyzed using recombinant *T. gondii* actin, *T. gondii* formin 1 seems to be a roughly tenfold weaker nucleator than formin 2, highlighting the importance of using homologous actin in functional assays (Skillman *et al.*, 2012 ▶). Like other characterized formins, the *Plasmodium* formin 1 FH2 domain depends on dimerization mediated by its so-called lasso region for its nucleation activity on both α-actin and recombinant *Plasmodium* actins (Ignatev *et al.*, 2012 ▶). Importantly, the presence of the rudimentary FH1 domain increases the elongation rate of muscle actin filaments more than the FH2 domain alone. Yet, profilin does not cooperatively further increase the rate together with the FH1 domain, but instead seems to compete for monomers with it. It thus needs to be investigated whether the formin 1 FH1 domain can bind to actin monomers without profilin as a mediator.

Many unanswered questions still remain, especially regarding the interaction between apicomplexan formins and profilins. In particular, the *Plasmodium* formin FH1 domains contain very few prolines compared with canonical FH1 domains. In addition, apicomplexan profilins bind polyproline peptides weakly (Daher *et al.*, 2010 ▶; Kursula, Kursula, Ganter *et al.*, 2008 ▶). In *Plasmodium*, formin 2, with at least two putative profilin-binding sites, is a likely candidate to take part in profilin-mediated actin elongation, while the role of formin 1 seems to be independent of profilin. As the number of profilin-binding sites generally correlates with the capacity to increase elongation speed (Paul *et al.*, 2008 ▶), even the formin 2/profilin combination would seem to be equipped to provide only a moderate acceleration in polymerization.

### 5.2. Coronin   

Coronins are a group of F-actin binders characterized by an N-terminal seven-bladed β-propeller domain and an interconnecting conserved region followed by a unique region and a C-terminal coiled-coil domain (Chan *et al.*, 2011 ▶). Coronins are classified into four different types. Types I and II differ mostly in their unique regions and only slightly in their β-propeller domains, but are otherwise structurally similar. Type III coronins are formed by a duplication of the N-terminal domains and the unique region and lack the coiled-coil region (Chan *et al.*, 2011 ▶). Type IV coronins are fused to villin headpiece domains (Eckert *et al.*, 2011 ▶). Apicomplexa have singular coronins that resemble type I coronins. We will therefore refer to other type I coronins for classical coronin functions.

There are currently four published structures of coronins in the PDB: two are structures of mouse coronin 1A without the coiled-coil region (PDB entries 2aq5 and 2b4e; Appleton *et al.*, 2006 ▶), one is a structure of the coiled-coil region of the same protein (PDB entry 2akf; Kammerer *et al.*, 2005 ▶) and the fourth is of the N-terminal domain and the conserved region of *T. gondii* coronin (PDB entry 4ozu; Salamun *et al.*, 2014 ▶). The N-terminal domain in all of these structures contains five complete and two incomplete WD40 sequence repeats, which form a characteristic β-propeller structure (Fig. 4 ▶ *e*). This WD40 domain is followed by a conserved region that folds underneath the β-propeller (if the actin-binding face is considered to be the top face of the structure) and provides stabilizing interactions to the WD40 domain. The unique and coiled-coil regions form an extended structure that is mainly involved in oligomerization but also in other functions related to actin dynamics (Spoerl *et al.*, 2002 ▶; Gandhi *et al.*, 2009 ▶). The function of coronins revolves around their ability to bind filamentous actin and confer effects through this binding alone or through interactions with other proteins. Yeast coronin 1, a type I-like coronin, inhibits the depolymerization and severing of ATP–actin filaments by cofilin, while a deletion mutant lacking the coiled-coil domain instead increases the severing effect of cofilin (Gandhi *et al.*, 2009 ▶). On the other hand, full-length coronin 1 alone is able to accelerate ADP–actin polymerization, likely by severing, suggesting that it has different effects on actin in different nucleotide states. Moreover, the C-terminal coiled-coil domain harbours a secondary actin-binding site and inhibits the severing effects of cofilin (Gandhi *et al.*, 2009 ▶), although contradictory reports suggest no binding of fragments of the coiled-coil domain of coronin 1 to F-actin (Liu *et al.*, 2011 ▶). The binding of the yeast coronin 1 WD40 domain to F-actin in ADP and ADP–P_i_ states has recently been studied using cryo-EM at an intermediate resolution of ∼8 Å (Ge *et al.*, 2014 ▶). The binding site for coronin on the actin filament in the ADP–P_i_ state clashes with the experimentally determined binding site for cofilin, while in the ADP state coronin is rearranged into a conformation that does not prevent cofilin binding. Thus, the different effects of coronin and cofilin on F-actin are explained by different binding modes of coronin to actin in different nucleotide states.

The sequence similarity between the two apicomplexan coronins, mouse coronin 1A, and yeast coronin 1 is approximately 30%, covering the WD40 and the conserved region, while there is next to no homology in the C-terminal regions. Even the *Plasmodium* and *T. gondii* coronins are very dissimilar in this region in terms of size, sequence identity and properties such as the presence of phosphorylation sites. *P. falciparum* coronin harbours as many as 11 phosphorylation sites in its C-terminal region (19 in total), while *T. gondii* coronin seems to have none (Treeck *et al.*, 2011 ▶). These dissimilarities may prove to confer specific functions for the coronins of different Apicomplexa and therefore warrant more investigation.

Proteolytic cleavage of coronins from the C-terminus might play a role *in vivo*. Appleton *et al.* (2006 ▶) found that full-length mouse coronin 1A produced recombinantly in insect cells is cleaved approximately 30–50 residues from the C-terminus, at the border of the WD40/conserved and unique/coiled-coil domains. Similar results with recombinant full-length *T. gondii* and *P. falciparum* coronins have been observed *in vitro* (J. Kallio, unpublished work) as well as in whole-cell lysates of *T. gondii* tachyzoites probed with an antibody recognizing N-terminally myc-tagged *T. gondii* coronin (Salamun *et al.*, 2014 ▶). It remains to be seen whether this proteolytic cleavage represents a mode of regulation for coronins (Appleton *et al.*, 2006 ▶; Gandhi *et al.*, 2009 ▶; Salamun *et al.*, 2014 ▶).

As the apicomplexan actins seem to respond uniquely to the state of the bound nucleotide, it would be fruitful to investigate how coronins respond to these states. Analogously to other type I coronins, apicomplexan coronins could differentiate between different nucleotide states of actin in the filament, with possibly a different effect for each nucleotide state. Type I coronins prefer ATP–actin or ADP–P_i_–actin to ADP–actin (Cai *et al.*, 2007 ▶; Gandhi *et al.*, 2009 ▶), and recent biochemical data suggest that this may also be the case for at least *T. gondii* coronin (Salamun *et al.*, 2014 ▶). However, the affinities and binding modes of *T. gondii* and *Plasmodium* coronins to ATP–actin and ADP–actin remain to be elucidated.

### 5.3. Capping proteins   

Filament capping is an essential process in the leading-edge formation of motile cells. The capping of individual filaments, especially branches of growing actin networks in the leading edge, is important for the conservation of energy, as it prevents the wasteful use of actin monomers in branches that are not producing force effectively. Based on the current knowledge of apicomplexan actin, it is unlikely that heavily branched networks exist, as many branching-inducing proteins such as the Arp2/3 complex are missing. Regardless of this implication, apicomplexan genomes encode genes for the α- and β-subunits of capping protein (CP), which provides barbed-end capping functions in the majority of actin-expressing organisms.

CP is classically a heterodimer composed of α- and β-subunits and binds actin filaments at the barbed end, preventing polymerization as well as depolymerization. Structurally, CP is very well characterized in higher eukary­otes and a great deal is known about its binding to the filament barbed end. The overall structure of CP is elongated and contains a distinctive, large antiparallel β-sheet that spans the whole of the longest axis of the heterodimer. This central β-sheet is covered on the other side by one N-terminal three-helix bundle per subunit and a cluster of four (α-subunit) or three (β-subunit) β-strands, and on the actin-binding side by one long and one short α-helix per subunit (Yamashita *et al.*, 2003 ▶). Additionally, the flexible, amphipathic C-terminal helices, termed the α- and β-tentacles, of both subunits are implicated in binding to barbed ends.

Homology modelling of the *Plasmodium* CP α- and β-subunits (Fig. 4 ▶ *f*) suggests that the overall fold is similar to classical CPs, with an insertion at the beginning of the central β-sheet of the α-subunit and an extension of the C-terminal helix in the β-subunit (Ganter *et al.*, 2009 ▶). However, experimental structural data on the nature of the insertions in CP is still missing. Similar insertions in apicomplexan actin regulators have been described before for, for example, profilin (Kursula, Kursula, Ganter *et al.*, 2008 ▶). Sequence alignments of putative CP subunits in *T. gondii* show a larger variation, with more prominent insertions in the central β-sheet (α-subunit; >100 residues) as well as more moderate insertions in the N-terminus (α- and β-subunits), the short helix region (β-subunit) and the β-tentacle. Because of these insertions, it is not possible to deduce the exact dimerization mode of the apicomplexan CPs. In *Plasmodium*, the CP β-subunit is not essential for the pathogenic blood stages and its deletion causes mild effects on ookinete motility (Ganter *et al.*, 2009 ▶). This raises the question whether the two subunits can function alone in at least certain life-cycle stages and whether heterodimerization or homodimerization of these proteins occurs.

## 6. Myosin A and gliding-associated proteins   

In the currently prevailing linear motor model (Fig. 1 ▶), the connection between actin filaments and the IMC is achieved through a complex of at least five proteins: (i) MyoA, a class XIV myosin unique to the Apicomplexa, (ii) one or more myosin light-chain homologues, (iii) gliding-associated protein (GAP) 50, (iv) GAP45 and (v) GAP40. MyoA is an unconventional myosin that lacks a tail domain but has a neck that binds to the light chain(s) (Heaslip *et al.*, 2010 ▶). The *Plasmodium* MyoA light chain MTIP binds a tail peptide of MyoA and open and closed conformations have been described (Bosch *et al.*, 2006 ▶; Bosch, Turley *et al.*, 2007 ▶). MTIP and its homologue in *T. gondii*, MLC1, have a long N-terminal extension that at least partly fulfills the role of the traditional myosin tail domain in linking MyoA to the IMC (Bosch *et al.*, 2006 ▶; Heaslip *et al.*, 2010 ▶). In *T. gondii*, a second light chain, ELC1, has also been described (Nebl *et al.*, 2011 ▶), and both MLC1 and ELC1 are required for fast motility along canonical actin filaments (Bookwalter *et al.*, 2014 ▶). The glideosome components are regarded as putative drug targets because of their uniqueness in apicomplexan parasites, and host-cell invasion is inhibited by a MyoA C-terminal peptide that binds to the light chain (Bosch *et al.*, 2006 ▶; Kortagere *et al.*, 2010 ▶; Thomas *et al.*, 2010 ▶).

GAP50 has a single C-terminal transmembrane helix that spans the outer leaflet of the IMC and a soluble globular domain that resides beneath the two IMC leaflets (Gaskins *et al.*, 2004 ▶; Johnson *et al.*, 2007 ▶; Bosch *et al.*, 2012 ▶). The structure of the GAP50 soluble domain has been determined and shows an αββα fold, belonging to the family of calcineurin phosphatases (Bosch *et al.*, 2012 ▶; Fig. 1 ▶ *b*). GAP45 is a membrane-associated protein that is likely to have little globular folded structure and is linked to the parasite plasma membrane and the IMC *via* lipid modifications to its N- and C-termini, respectively (Frénal *et al.*, 2010 ▶). The C-terminal region of GAP45 also interacts with the MyoA–light chain complex, recruiting it to the IMC. GAP40 is the most recently identified component of the glideosome (Frénal *et al.*, 2010 ▶). It is a seven-helix integral transmembrane protein and its function is still largely unknown.

In light of recent results showing that many of the glideosome components, including actin and MyoA, can be knocked out without completely abolishing the ability of the parasites to move and invade, the components, structure and role of the glideosome need re-evaluation (Andenmatten *et al.*, 2013 ▶; Egarter *et al.*, 2014 ▶). It is possible that the mechanism of gliding is different to what has been imagined, or that alternative mechanisms exist that can compensate for the loss of several components of this machinery crucial for parasite survival. In fact, such plasticity between gliding-associated proteins and myosins has recently been reported in *T. gondii* (Frénal *et al.*, 2010 ▶, 2014 ▶). The recently described successful heterologous expression and purification of functional *T. gondii* MyoA with its light chains may pave the way for a structural and biochemical understanding of the mechanism of force generation in the parasite actin–myosin motor (Bookwalter *et al.*, 2014 ▶).

## 7. Link to the plasma membrane and the host cell   

On the plasma-membrane side, the actin–myosin motor is linked to surface adhesin molecules of the thrombospondin-related adhesive protein (TRAP) family in *Plasmodium*, the micronemal protein (MIC) 2 in *T. gondii* or apical membrane protein (AMA) 1 (Buguliskis *et al.*, 2010 ▶; Cowman *et al.*, 2012 ▶). The adhesins contain variable extracellular adhesive domains that recognize the host cell, a transmembrane domain and an acidic cytoplasmic tail that has a conserved tryptophan residue at the C-terminus (Carruthers & Tomley, 2008 ▶). Several adhesins have long been thought to be connected to the actin filaments *via* aldolase as a bridging molecule (Buscaglia *et al.*, 2003 ▶; Jewett & Sibley, 2003 ▶). Aldolase binds to the cytoplasmic tails of TRAP and several other adhesins and increases actin polymerization *in vitro* (Diaz *et al.*, 2014 ▶). The structure of the *Plasmodium* TRAP cytoplasmic tail in complex with aldolase has also been determined (Bosch, Buscaglia *et al.*, 2007 ▶). However, it was recently shown that in *T. gondii* mutations to AMA1 that disrupt aldolase binding do not affect host-cell invasion (Shen & Sibley, 2014 ▶). Thus, the role of aldolase in motility, invasion and actin dynamics also has to be revisited.

## 8. Outlook   

**Figure 5:**
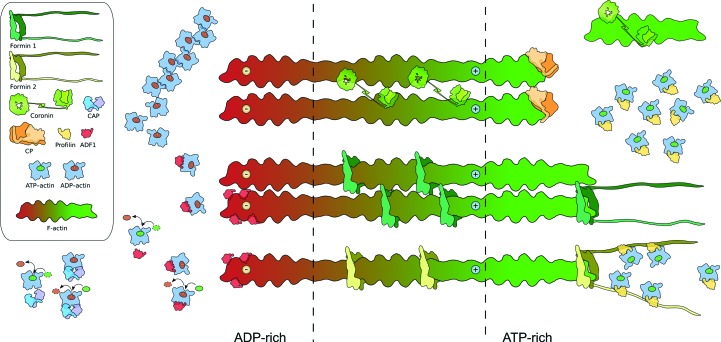
A schematic presentation of the roles of different actin regulators in the context of apicomplexan actin filaments. The green colour in the actin filaments indicates ATP-rich filaments and the red colour represents ADP-rich filaments. At the ATP-rich end, profilin sequesters ATP–actin monomers and provides them to formins (most likely formin 2), while CP caps growing filaments and coronin stabilizes short, newly formed filaments. In the middle region, coronin and formins bind filaments and possibly cross-link or bundle them into larger assemblies. At the ADP-rich end, ADF1 accelerates depolymerization and promotes nucleotide exchange on the monomers. CAP accelerates nucleotide exchange and sequesters actin monomers. Free ADP–actin monomers removed from the pointed end or having gone through spontaneous ATP hydrolysis in the monomeric state assemble into short oligomeric structures. It is not clear whether these oligomers are helical or linear polymers.

The linear motor model of apicomplexan gliding motility has been an elegant working model for around two decades. However, despite most of the components being identified and also characterized to some extent, we are still far from a mechanistic understanding of how the force for gliding is actually generated. The number of essential components in the gliding machinery is small, which makes the system attractive for structural studies and makes up a model system for the minimal requirements for a functional actin-based motor. Such a system seems feasible for a small-scale ‘structural systems biology’ approach with the goal of creating a detailed molecular picture of the structure and function of the entire polymerization machinery. In the last few years, structural and biochemical information has been accumulating, especially on *Plasmodium* actin and its regulatory proteins, providing clues to their roles (Fig. 5 ▶). Now, with most of the components at hand, it is time to start looking into larger complexes and dynamics of the entire system. On the other hand, the recombinant proteins and high-resolution structures at hand should be used for their evaluation as potential drug targets. Many of the structural and functional differences in these proteins from their human counterparts warrant such efforts.

The parasite actin–myosin motor is also interesting from the evolutionary point of view. Even actin itself differs markedly from all other actins characterized to date and shares some conserved features, for example with the bacterial actin homologue MreB (Vahokoski *et al.*, 2014 ▶). An interesting question is whether or not there is polarity in apicomplexan actin filaments. For canonical actins polarity is paramount, but apicomplexan actins may not share this necessity. To date, proteins interacting with the pointed end of apicomplexan actin filaments have not been identified, while the very low or nonexistent apparent critical concentration (Skillman *et al.*, 2013 ▶) and the unconventional link between ATP hydrolysis and polymerization (Vahokoski *et al.*, 2014 ▶) give grounds to speculate that the mechanism of growth may be very different in apicomplexan actins. However, further biophysical and structural evidence needs to be gathered before any tangible models for actin polymerization in Apicomplexa can be generated.
